# Data-Driven Molecular Dynamics: A Multifaceted Challenge

**DOI:** 10.3390/ph13090253

**Published:** 2020-09-18

**Authors:** Mattia Bernetti, Martina Bertazzo, Matteo Masetti

**Affiliations:** 1 Scuola Internazionale Superiore di Studi Avanzati (SISSA), via Bonomea 265, I-34136 Trieste, Italy; mbernett@sissa.it; 2 Computational Sciences, Istituto Italiano di Tecnologia, via Morego 30, I-16163 Genova, Italy; martina.bertazzo@iit.it; 3 Department of Pharmacy and Biotechnology, Alma Mater Studiorum—Università di Bologna, via Belmeloro 6, I-40126 Bologna, Italy

**Keywords:** machine learning, dimensionality reduction, reaction coordinates, collective variables, Markov state models, maximum entropy principle, experimental data

## Abstract

The big data concept is currently revolutionizing several fields of science including drug discovery and development. While opening up new perspectives for better drug design and related strategies, big data analysis strongly challenges our current ability to manage and exploit an extraordinarily large and possibly diverse amount of information. The recent renewal of machine learning (ML)-based algorithms is key in providing the proper framework for addressing this issue. In this respect, the impact on the exploitation of molecular dynamics (MD) simulations, which have recently reached mainstream status in computational drug discovery, can be remarkable. Here, we review the recent progress in the use of ML methods coupled to biomolecular simulations with potentially relevant implications for drug design. Specifically, we show how different ML-based strategies can be applied to the outcome of MD simulations for gaining knowledge and enhancing sampling. Finally, we discuss how intrinsic limitations of MD in accurately modeling biomolecular systems can be alleviated by including information coming from experimental data.

## 1. Introduction

The idea of exploiting computers and information technology for assisting the drug discovery process goes back to the early 1960s when the pioneering works by Corwin Hansch and Toshio Fushita laid the ground for quantitative structure-activity/property relationship (QSAR/QSPR) models [1,2]. Since then, several computational approaches have flourished and, as soon as adequate computational resources have reached widespread availability, computer-aided drug discovery (CADD) has become a valuable asset for both academia and industry [3]. Nowadays, different kinds of computational methods are virtually employed in all realms of science and, in the field of medicinal chemistry, they are widely recognized as an integral part of any modern drug discovery endeavor [4]. Many of these approaches do rely on the availability of experimental data to draw hypotheses, identify patterns, and make inferences (inductive learning) [5]. Thanks to recent advances in experimental techniques, like high-throughput screening and array measurements, among others, we are today in the position of disclosing the full potential of these computational tools [6]. Nevertheless, mining and integrating large-scale datasets coming from different sources is far from trivial. This issue is a declination of the well-known problem of big data, which refers to an overwhelming amount of information (“big” in terms of volume and diversity) that challenges the possibility of taking advantage of it [7]. It is a common perception that the current resurgence of artificial intelligence (AI) methods, including brand new techniques like deep learning (DL) algorithms, will ultimately provide a suitable framework to address this task [8,9].

**Figure 1:**
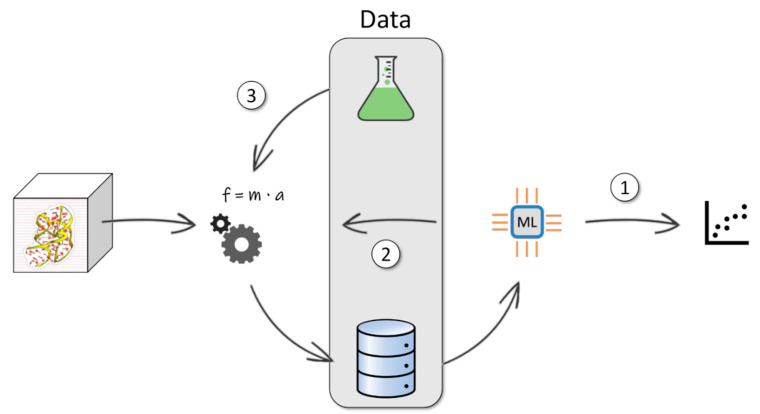
Pictorial representation of data exploitation in molecular dynamics (MD) simulations. Note that the source of data can be either computational (the very output of MD simulations, paths “1” and “2”) or experimental (path “3”). Path “1” refers to the use of machine learning (ML) methods for the conventional analysis step performed a posteriori once the MD data have been generated. Path “2” depicts a loop where ML methods enter during the simulations to inform subsequent MD runs (specifically consisting of simulation runs, data generation, and ML-based data analysis). This loop can be either discontinuous (MD/ML resampling) or seamless (on-the-fly MD/ML).

Over the past decade, another class of computational methods has gained increasing popularity in the field of drug discovery and development. These are based on the application of fundamental theories to predict the properties of materials and can be ascribed to deductive learning [5]. Molecular dynamics (MD) is one of such approaches that stem from the possibility of simulating the temporal evolution of systems (in jargon, the trajectory) based on a microscopic description thereof [10]. Given that a suitable representation of intra- and inter-molecular forces is provided (the so-called force field) and thus interactions between biomolecules are properly modeled, MD allows investigating molecular-level mechanisms and extracting relevant observables related to the process under investigation. Protein–ligand (un)binding mechanisms (i.e., “dynamic docking”), binding free energies, and even kinetic constants are examples of useful outcomes for drug design when MD simulations are applied to pharmaceutically relevant systems [11,12,13]. It is therefore not surprising that MD methods are nowadays routinely employed in drug discovery for complementing experiments and orthogonal computational techniques like molecular docking and virtual screening [14,15]. Notwithstanding their descriptive and predictive power, MD historically suffered from two main drawbacks: the limited accuracy of force fields and the short length of simulations compared to the real physical time required to observe the investigated biological events. Thanks to the advancements in high-performance (HPC) and/or distributed computing, the second issue is alleviating, as the gap between experimental and computational timescales is constantly decreasing. The straight drawback, however, is that trajectories and output data, in general, are steadily growing in size, calling for adequate strategies for extracting relevant information when the big data regime approaches. Even from this standpoint, AI-based methods are optimally equipped to cope with such increased complexity. It is worth highlighting that machine learning (ML) has long been adopted by the MD community to analyze simulations of biomolecular systems [16]. Nevertheless, not only today are these approaches becoming routine for processing MD-derived data, but the growing awareness of their potential is also boosting their development for gathering insight in an automated fashion, for informing subsequent simulations, and even for analyzing and guiding the dynamics in a seamless way (see Figure 1, paths “1” and “2”). Prominent parallel processing strategies are also starting to be explored for dealing with large-scale MD data analysis [17]. These include efficient tools such as Hadoop, an open-source implementation of MapReduce [18], which, differently from classical HPC frameworks with dedicated storage nodes, are instead based on localized storage on the compute nodes. By leveraging on such architectures that facilitate access to data, the performance of analysis algorithms can be remarkably improved. While such applications to MD-generated data are still in their infancy, they have the potential to become remarkable instruments as hardware resources lead towards the big data domain. A detailed discussion of this topic is out of the scope of the present review and will thus not be covered.

The big data problem refers not only to the quantity and diversity of data but also to their rate of production and trustworthiness of their sources (also known as “veracity”). In this context, it is worth highlighting that a class of emerging analysis methods is explicitly concerned with the combination of MD-derived data and experimental information to deal with the above-mentioned force field issue. Specifically, such strategies aim at reducing systematic inaccuracies due to the limitations of the force field used to model the biomolecular systems [19]. Albeit not strictly inside the ML domain, these approaches represent an innovative way of making sense from MD trajectories or guiding new simulations by enforcing the agreement with available experimental knowledge (see Figure 1, path “3”).

In this review, we summarize the different classes of ML methods applied to the analysis of MD trajectories (hereafter referred to as MD/ML approaches) holding great potential in the field of biomolecular simulations. Here, we stress that some of these methods have been widely employed for decades, like clustering and principal component analysis (PCA), while others have been introduced only very recently, and their application to real-life drug discovery cases is yet to come. Finally, we provide an overview of the emerging methods to include experimental information in MD simulations for better modeling biomolecular systems.

## 2. Learning from Molecular Dynamics Trajectories

Molecular dynamics is concerned with the time evolution of systems under the classical laws of motion. The integrator is at the very heart of any MD engine, as it takes care of solving Newton’s equations iteratively for discrete steps in time (i.e., the time-step). To satisfy a stable and accurate integration, one needs to choose the time-step to be significantly smaller than the characteristic oscillations of the system under consideration, typically 1–2 fs. This has two important implications. First, reaching experimentally relevant timescales for the events one wishes to investigate (micro-, milli-seconds, and possibly more) becomes a formidable computational task [15]. Second, for MD to be informative, the trajectory must be saved to a disk at a sufficiently high pace, thus generating a huge amount of data that requires further analysis. To deal with the former issue, apart from mere technological advances, several and highly diversified methods have been developed over the years. A large class of such methods relies on the notion of reaction coordinates (or collective variables, CVs), which are functions of the atomic positions whereby the investigated event is accelerated through external biases in the form of additional forces or potentials. The computation of most common CVs can be already found implemented either in MD codes, such as NAMD [20,21] and AMBER [22], or in dedicated software such as PLUMED [23]. Among the most popular approaches exploiting CVs, we mention umbrella sampling [24], steered dynamics [25,26], adaptive biasing force [27], and metadynamics [28,29]. Here, for simplicity, we refer collectively to these methods as “biased sampling” and we redirect the interested reader to more specific reviews for further details [30,31]. Biased sampling methods can be highly informative because not only do they speed up the observation of “rare events” but, under proper simulative conditions, they also allow to retrieve the underlying free energy landscape. Unfortunately, choosing the proper CVs is not always straightforward, and much chemical intuition and/or trial-and-error procedures are often required [32].

Concerning the second issue, namely coping with a big data scenario, the application of ML methods can be of great benefit to the analysis of MD trajectories (Figure 1, path “1”). Furthermore, ML can also be integrated into MD protocols to optimize the production of MD trajectories. In particular, concerning the latter point, ML methods can help in identifying the CVs required for biased sampling simulations (Figure 1, path “2”). Notably, this can either be done subsequently, that is running biased sampling of CVs after they are identified from the analysis of one or more explorative MD simulations (MD/ML resampling), or via on-the-fly protocols (on-the-fly MD/ML). On-the-fly learning and sampling approaches probably represent the most elegant way of combining MD with ML, as they relieve the user of rather subjective choices and often tedious rounds of simulation and analysis. We note that on-the-fly MD/ML can be also declined in what we call here “guided sampling”. This class of methods bears some similarities with adaptive sampling procedures like weighted ensemble [33,34], among others. In particular, they share the feature of taking care of launching and controlling repeated sequences of multiple MD simulations in an automated fashion. In addition to assisting the identification of CVs, in this case, ML methods help in optimally identifying the starting states for each sequence of MD runs. In practice, the states are drawn so as to “guide” the system towards undersampled or even previously unexplored regions of configurational space, thus achieving a wider exploration without the need of introducing external biases.

ML methods are generally classified into two broad categories: supervised and unsupervised learning (Table 1) [35]. While unsupervised learning deals with the identification of patterns among data, the ultimate goal of supervised learning is to disclose the relationships (if any) between dependent and independent variables. In this case, the learning procedure is carried out by partitioning the available dataset into two chunks: the training and the test set. The former is employed to train the model, while the latter is exploited to validate its performance in predicting the dependent variables for the subset of data that was not considered during training. It is important to recognize that, in the context of the analysis of MD trajectories, ML methods most often take as input convenient representations of the configuration of the system over time, rather than bare atomic coordinates [36]. These representations must satisfy symmetry invariances (rigid body translations and rotations as well as permutations of identical atoms) and can be described as a vector of features, like dihedral angles, or CVs in general, like contact maps, and so on. Notably, in the ML jargon, this process of mapping the Cartesian coordinates into the space of selected features of interest is typically referred to as “featurization” [8].

### 2.1. Unsupervised Learning Methods

#### 2.1.1. Clustering and PCA: The Grand Old Tools of Trajectory Analysis

**Figure 2:**
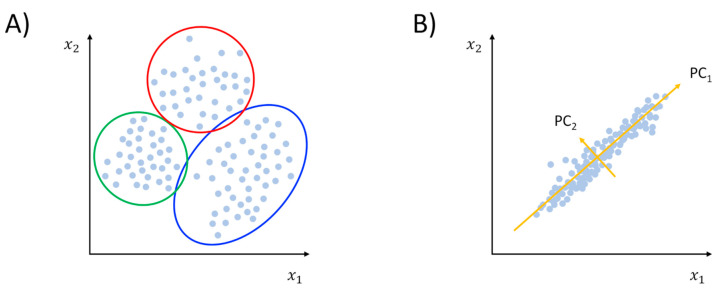
Pictorial representation of the unsupervised learning class of methods: cluster analysis (panel (**A**)) and dimensionality reduction (panel (**B**), principal component analysis (PCA) is displayed as a representative example).

Unsupervised learning can be distinguished into clustering and dimensionality reduction (Figure 2). A more technical discussion of the use of these methods in the context of MD simulations can be found in an excellent review by M. Ceriotti [36]. Briefly, clustering methods attempt to partition input data into classes where members of the same group can be considered more similar between them than members belonging to different groups (Figure 2A). To implement the concept of similarity, one must define a way to measure distances in the feature space. Typically, the pairwise atomic root-mean-squared-deviation (RMSD) is used to this aim:

(1) $$RMSD_{ij}=\sqrt{\frac{1}{N}\sum_{k=1}^{N}{\left(x_{k}^{i}-x_{k}^{j}\right)}^{2}},$$

where *N* is the number of atoms in the summation, and $x_{k}^{i}$ and $x_{k}^{j}$ are features of the system in two different configurations, *i* and *j*, sampled in an MD trajectory. When dealing with MD simulations, a common practice is to use atomic coordinates as input features, but other metrics can be envisioned. In this context, cluster analysis is very helpful for identifying the metastable states (i.e., local free energy basins) explored during the dynamics and it represents a popular way to analyze MD trajectories [37]. It is out of the scope of the present work to review such kind of approaches, as the variety of methods and the extent of applications would require an entire article to fully describe them [38]. We limit ourselves by mentioning an on-the-fly MD/ML approach introduced by Tribello et al., where the sampling is biased making use of a sophisticated clustering procedure (Gaussian mixtures) as a central ingredient [39]. The method, dubbed reconnaissance metadynamics, accelerates the dynamics through a repertoire of CVs that are expected to provide the best local approximation of each free energy well that is visited during the simulation. Cluster analysis is performed at regular intervals on the collected trajectory, and the identified clusters are then exploited for tuning a one-dimensional CV that will be used for biasing the dynamics until the next round of analysis [39].

While clustering helps in identifying groups of configurations basing on feature similarity, dimensionality reduction (or manifold learning) represents a variety of methods whose aim, as the name suggests, is to reduce the dimension of the feature vectors [36,40]. In other words, dimensionality reduction methods seek to find a low-dimensional (low-d) manifold embedded in the high-dimensional (high-d) space represented by the input data structure [32]. The procedure is rooted in the possibility of spotting redundancies and correlations that are often found in large data samples [35]. Once the low-d space is obtained, the interpretability of data is in general improved. One should keep in mind that such simplification always comes with a certain degree of information loss. Thus, finding an optimal tradeoff between the two can be difficult in some cases. The forerunner of all dimensionality reduction methods is certainly principal component analysis (PCA), which has become a popular MD analysis tool under the moniker of “essential dynamics” [41]. Technically speaking, PCA provides a linear transformation of the feature vectors in a way that best captures the variance of data. The outcome of the dimensionality reduction is therefore a set of eigenvectors (or principal components, PCs) ranked by the decreasing fraction of the total variance explained (eigenvalues, see Figure 2B). While in principle this can be applied to all sorts of features, it is common practice to apply PCA to atomic coordinates. Specifically, eigendecomposition can be performed by diagonalizing the covariance matrix of atomic fluctuations, whose elements are

(2) $$C_{ij}=\langle x_{i}\cdot x_{j}\rangle ,$$

where $x_{i}$ and $x_{j}$ are the positional fluctuation vectors of atoms *i* and *j*, while the angle brackets denote the average evaluated over the entire simulation (ensemble average). We note, in passing, that in the one-dimensional case, the elements of the normalized covariance matrix ($C_{ij}=\langle x_{i}\cdot x_{j}\rangle /\sqrt{\langle x_{i}\rangle {}^{2}\langle x_{j}\rangle {}^{2}}$) correspond to the Pearson correlation coefficient (r) between the two variables.

From a practical standpoint, this powerful ML analysis tool is used to identify the correlated motions of proteins or biomolecules in general. This is especially relevant as the projection of the trajectory on the first few principal components allows a rather straightforward identification of metastable states and transitions among them without the need of resorting to a cumbersome visual inspection of configurations. Thus, PCA can be in principle employed to study mechanisms underlying conformational transitions, ranging from minor local rearrangements up to entire folding processes (but see below for caveats). As in the case of cluster analysis, even providing a partial list of the most recent applications of PCA in the context of MD analysis would be unfeasible. We rather highlight here that the principal components extracted from MD simulations can be thought of as a set of CVs, and as such, they can be used for biased MD/ML resampling. This idea has been pursued in a form of restrained dynamics (“essential dynamics sampling” [42]) and metadynamics as well [43]. We note, however, that while PCs can be considered as good order parameters whenever they allow to clearly distinguish among the most relevant states, for a number of reasons, they are not also necessarily good CVs for biased sampling. Indeed, despite its conceptual simplicity and ease of use, which pushed the implementation in several MD analysis tools over the years [22,23,44,45,46,47,48], PCA is not free from limitations. A technical drawback is related to the fact that PCA is typically fed with the Cartesian coordinates of a given subset of atoms (usually the backbone or Cα atoms in the case of proteins). In order to remove irrelevant motions like rigid body translations and rotation, it is customary to align the frames of the trajectory on a reference structure, which is often times the starting configuration or an average conformation. Thus, the results of the dimensionality reduction are somewhat dependent upon the choice of both the reference structure and the atoms used for finding the optimal alignment. A possibility to bypass this problem is choosing a different feature space, like internal coordinates [32]. For example, PCA on dihedral angles can be performed [49,50], and this approach has been recently used by Ferraro et al. to rationalize the change in the efficacy of a series of congeneric modulators of the dopamine D3 receptor [51].

A more elegant choice over PCA, however, is taking advantage of multidimensional scaling (MDS), a distinct, but somehow related ML tool. Differently from PCA, MDS (sometimes also referred to as principal coordinate analysis [52]) operates directly on pairwise distances between conformations (like the RMSD), thus avoiding the optimal alignment problem. Hence, in MDS, the problem can be reformulated as finding the embedding that best preserves the distances evaluated in the high-d space. MDS comes in two flavors: the original algebraic formulation, also known as “classical” MDS, and an optimization procedure through iterative algorithms (distance scaling, or “metric” MDS). The idea behind classical MDS is to transform the distance matrix into an inner product matrix that can be further diagonalized as in PCA. The ground for this reasoning is that, in Euclidean space, distances ($D_{ij}$) are related to inner products as follows:

(3) $$D_{ij}^{2}={\left|x_{i}-x_{j}\right|}^{2}={\left|x_{i}\right|}^{2}+{\left|x_{j}\right|}^{2}-2\langle x_{i}\cdot x_{j}\rangle .$$

Thus, after a procedure called double centering which takes care of the fact that inner products depend on the origin while distances do not, by inverting this relationship, one obtains the desired inner product matrix [52]. The set of obtained eigenvectors has much the same significance as in PCA, even though MDS is considered more general as it can also be applied to non-Euclidean high-d spaces [32]. Conversely, still under the assumption of a linear projection, in the simplest form of metric MDS, one rather optimizes the loss function [36]:

(4) $$l_{ij}^{2}=\sum_{ij}{\left(D_{ij}-d_{ij}\right)}^{2},$$

where $D_{ij}$ and $d_{ij}$ are the distances in the high- and low-d spaces, respectively. An important requirement of dimensionality reduction methods is the ability to map new high-d data points into a previously obtained embedding. This is the so-called “out-of-sample” problem that affects MDS and related methods. We note that PCA is devoided from this limitation, as new data points can be easily projected on to the PCs using the same linear transformation employed to carry out the dimensionality reduction. To the best of our knowledge, the first application of linear MDS in the analysis of MD trajectories was reported by Troyer and Cohen as early as 1995 [53]. More recently, Pisani et al. reported on an interesting application of MDS for mapping the conformations explored by the CDK2 protein kinase during MD simulations [54]. Notably, the embedding was constructed using a pool of experimentally derived structures, and an appropriate out-of-sample extension was devised to map the trajectories points on the previously derived low-d space [54].

Another quite serious problem with PCA is that, by construction, principal components only provide a linear mapping of the high-d space of input data. Thus, meaningful results can only be obtained if input data are linearly correlated. While at the bottom of free energy wells the motion of biomolecules might satisfy the quasi-harmonic approximation, in general, this assumption is no longer valid in the proximity of transition state regions. This means that studying complex and highly non-linear rearrangements like protein folding, while technically feasible, can lead to arguable results. For the same reason, while PCA can be used to gain mechanistic insight at a qualitative level, it should not in general be used for extracting rates related to the process under investigation. The same reasoning applies to linear MDS.

#### 2.1.2. Beyond Linear Dimensionality Reduction

The concept of non-linearity in dimensionality reduction methods can be implemented in several ways. Perhaps, the simplest way to achieve this is through kernel PCA (kPCA) that can be thought of as a generalization of PCA. The idea behind kernel methods is to exploit a non-linear transformation of the input data into some feature space Φ(x) of higher dimensionality, with the hope of finding linear correlations in this new space. In particular, the kernel is a function that represents the inner product in the feature space:

(5) $$k\left(x_{i},x_{j}\right)=\Phi \left(x_{i}\right)\cdot \Phi \left(x_{i}\right).$$

By diagonalizing the matrix whose elements correspond to this product, one obtains principal components like in PCA, but in this case, one attempts to capture non-linearity in the high-dimensional space through the definition of the kernel itself. The advantage is that one does not need to explicitly compute the mapping function Φ(x), as the kernel matrix can be readily obtained by the input data using a polynomial, exponential, or sigmoid function. Indeed, the covariance matrix that is diagonalized in PCA can be considered as the simplest possible kernel function (the inner product). As an example, Antoniou and Schwartz successfully employed a polynomial kernel ($k\left(x_{i},x_{j}\right)={\left(x_{i}\cdot x_{j}\right)}^{n}$) to extract the CV describing the enzymatic reaction of the lactate dehydrogenase enzyme [55].

From a different standpoint, non-linearity can also be addressed within the MDS framework. Non-metric MDS (nMDS) can be considered as a form of non-linear MDS. Instead of attempting to preserve pairwise distances, it focuses on preserving their ranking in the high-d space. This is a useful approach to be considered when, rather than the exact value of the distance, the relationship among input data is thought to be more relevant. nMDS has been adopted to map the configurational space of the villin headpiece during folding trajectories that were previously generated through exceptionally long MD simulations for that time [56], and it was found to be superior to PCA and conventional cluster analysis [57]. Differently, Sketch Map is a non-linear metric MDS method introduced by Ceriotti et al. that seeks to preserve middle-ranged proximities in a way to collapse or amplify distances for points that are found below or above some characteristic length that is specific for the considered data structure (and that must be priorly assessed) [58]. The non-linear mapping is obtained through a modification of the loss function usually employed in metric MDS (Equation (4)):

(6) $$l_{ij}^{2}=\sum_{ij}{\left[F\left(D_{ij}\right)-f\left(d_{ij}\right)\right]}^{2},$$

where F and f are sigmoidal functions that are dependent on the choice of the aforementioned characteristic length. The rationale behind this approach is that, for complex molecular transitions like those observed in typical MD simulations, the noise in data due to thermal fluctuations will prevail in the proximity of minima, while undersampling will characterize the transitions between them [59]. Thus, by properly tuning the characteristic length, only the essential features of the high-d space will be preserved in the low-d embedding providing, as the name implies, a sketch of the entire energy landscape is visited by the system. Sketch Map comes with an efficient optimization strategy based on the selection of “landmark” (i.e., representative) points in the high-d space as well as a procedure to cope with the out-of-sample problem [58,59]. In the original implementation, Sketch Map was tested to reduce the dimensionality of the polyalanine-12 peptide using the 24-dimensional space of the backbone dihedral angles as input features [58]. Later, the same authors extended the methodology to carry out biased MD/ML resampling of a previously generated embedding in analogy with metadynamics (therefore, the method was called field-overlap metadynamics) [60]. Recently, among the several applications, Bellucci et al. applied Sketch Map to describe conformational changes of the 16–22 segment of the β-amyloid peptide upon binding to a gold surface [61].

**Figure 3:**
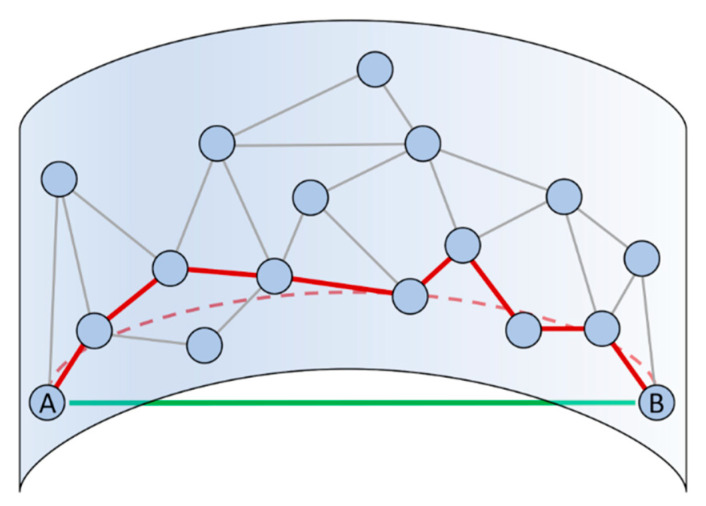
Schematic representation of the difference between the Euclidean and geodesic distance (green solid and red dashed lines, respectively) evaluated in a curved manifold. The network-based nearest neighbor approximation of the geodesic distance provided by Isomap is also shown (red solid lines).

Isometric feature mapping (Isomap) is another non-linear dimensionality reduction method that builds on MDS [62]. Rather than evaluating the Euclidean distance in the high-d space, Isomap estimates the geodesic distance, which is the distance along a straight line in a curved manifold. In particular, the geodesic distance is computed finding the shortest path through a network analysis performed on the high-d space (Figure 3) [62]. This approximation holds only in the limit of very dense sampling, and, when this requirement is fulfilled, the computation becomes highly inefficient. A variant of the original algorithm specifically designed for big datasets such as the output of MD simulations is the so-called scalable Isomap (ScIMAP) proposed by Clementi and co-workers [63]. ScIMAP alleviates the computational burden by choosing random landmark points and approximating the distances only between these points and the remaining ones, instead of calculating all the pairwise shortest paths [63]. This method has been used to map the conformational space and compute the conformational free energy of coarse-grain models of the Src homology domain 3 (SH3) protein and a 22-residues β-hairpin [63,64]. Isomap has also been extended for its use in the context of biased MD/ML resampling simulations. Notably, the out-of-sample problem and the requirement of a smooth mapping of the configurational space for computing biasing forces (i.e., the differentiability) have been elegantly bypassed by Spiwok and Králová [65] through a generalization of the path CVs previously introduced by Branduardi et al. [66]. These variables are nowadays referred to as “Property Maps” within the PLUMED community [23,67], but we highlight here that an Isomap embedding can also be used as a CV space through the more general “Smooth and Nonlinear Data-Driven CV” (SandCV) formalism developed by Hashemian et al. [68]. Finally, we mention that Isomap was recently used by Schuetz et al. to map the unbinding pathways of drug-like molecules from their target as obtained by high-effective temperature MD simulations [69]. The projection of these pathways onto the low-d space was then clustered using the Fréchet distance as a metric with the aim to gain insight on the unbinding mechanism of the considered molecules [69].

Aside from the above-discussed methods, the issue of non-linearity in dimensionality reduction can be addressed from another perspective. Starting from the limitations of conventional PCA, namely the linear approximation and the often overlooked problem that by construction only collinear motions can be detected as correlated (see Equation (2)), Lange and Grubmüller devised a generalized measure of correlation which rests on statistical mechanics arguments and information theory [70]. This generalized correlation coefficient ($r_{\mathrm{MI}}$) builds on Shannon’s mutual information (MI) between random variables, and, in analogy with the Pearson correlation coefficient r, it is conceived to return a value of 1 for fully correlated motions and 0 when no correlation is found [70]. By minimizing this MI measure in a procedure known as full correlation analysis (FCA), one obtains maximally uncoupled collective coordinates [71]. This is a form of independent component analysis (ICA, see below). Differently from PCA, where eigenvectors are ranked according to the amplitude of motion, the authors proposed a ranking based on the anharmonicity of the modes as assessed through the estimate of their negentropy [71]. For the investigated systems, FCA modes turned out to better describe conformational states and provided a better description of the transition pathway among basins than PCA, suggesting an improved ability to capture functional motions over linear methods [71]. As an example, FCA has been successfully employed to detect functional motions of the HIF-2α PAS-B domain that are possibly involved in assisting ligand (un)binding [72].

#### 2.1.3. Including Dynamical Information into Geometric Dimensionality Reduction

All the methods described in previous sections are based on (linear or non-linear) static properties of the high-d space. A step forward towards a complete mechanistic interpretation of the simulated events can be taken by including some dynamical information on the derivation of the reduced dimensionality space. Diffusion maps are one such example that attempt to preserve the dynamic proximity between configurations visited in the high-d space [73]. To do so, diffusion maps employ a Gaussian kernel (hence, it corresponds to a form of kPCA):

(7) $$k\left(x_{i},x_{j}\right)=e^{-\frac{RMSD_{ij}^{2}}{2\varepsilon^{2}}},$$

where ε is a characteristic timescale below which the metric can be considered a meaningful representation of the transition between the two configurations $x_{i}$ and $x_{j}$ [32,73]. With this definition of the kernel, the principal components correspond to the eigenvectors of the Fokker–Planck equation, and therefore they should provide a faithful interpretation of the dynamics of the system. Specifically, apart from the trivial zeroeth mode, the lower ranked diffusion coordinates (DC) correspond to the slowest collective motions of the system, and they can be used as CVs for further sampling [32,73]. Several variants of diffusion maps have been proposed over the years, including the locally scaled diffusion map (LSDMap) by Clementi and coworkers which is an extension of the original method specifically designed to cope with noisy data like that of MD simulations [74]. In particular, the authors introduced an algorithm for detecting the intrinsic dimensionality and the local timescale for each configuration, thus avoiding artifacts in the embedding arising from a uniform choice of the ε parameter [74]. LSDMap allowed the authors to extract well-behaved CVs and to estimate rates through Kramers’ rate theory [13,74]. Notably, the DCs captured by LSDMap are global coordinates representing the slowest modes of the entire molecule, while the definition of “local” information would be required for efficiently guiding the dynamics through on-the-fly MD/ML sampling. This idea is exploited in the so-called diffusion map-directed MD (DM-d-MD), where local DCs are estimated by periodically computing DCs, and restarting the simulation in the slowest mode [75]. In an extended version (extended DM-d-MD), the method was combined with a reweighting scheme ensuring the possibility to recover the Boltzmann distribution despite the artificial dynamics [76]. Another method that couples MD and on-the-fly non-linear manifold learning based on diffusion maps is intrinsic map dynamics (iMapD) developed by Chiavazzo et al. [77].

**Figure 4:**
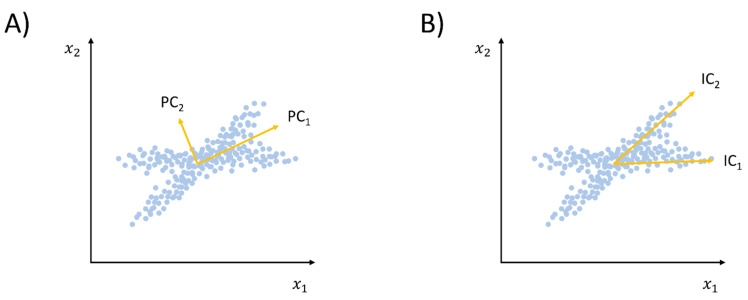
Difference between the components extracted through PCA (panel (**A**)) and a generic independent coordinate analysis (ICA) method (panel (**B**)). In specific cases, ICA provides a better description of the high-d data structure, as the eigenvectors identified are not necessarily restrained to the orthogonality relationship.

As already stressed, conventional PCA has several drawbacks, including the fact that only a linear correlation can be detected, and not entirely. Moreover, including dynamic information into the PCA framework is problematic, as PCs are not necessarily independent (even though they are orthogonal, see Figure 4). In order to overcome such limitations, Naritomi and Fuchigami introduced the time structure-based independent correlation analysis (tICA) method [78]. Differently from PCA, as already mentioned, ICA is an ML approach that attempts to extract components that are as statistically independent as possible. The tICA method differs from conventional ICA in that it also includes information on time dependency among the extracted eigenvectors. Accordingly, the usual time-independent covariance matrix of Equation (2) is replaced by a time-lagged covariance matrix:

(8) $$C_{ij}\left(\tau \right)=\langle x_{i}\left(t\right)\cdot x_{j}\left(t+\tau \right)\rangle ,$$

where τ is a given simulation lag time that must be properly chosen. By diagonalizing the time-lagged covariance matrix, one obtains eigenvectors (independent components, IC) that are no longer orthogonal to each other. Among the interesting properties of this formalism, we mention that the eigenvalues $\lambda_{i}$ provide information of the timescales of the associated IC, and in the special case of an autocorrelation function with a single exponential decay, the corresponding timescale $t_{i}$ can be expressed as

(9) $$t_{i}=-\frac{\tau }{\ln\lambda_{i}}.$$

Thus, not only can tICA be used to gain mechanistic insight regarding the motion, but it is also useful to get a rough estimate of the associated characteristic timescales [78]. For the analysis to be robust, however, it is critical to choose the lag time appropriately, as any IC with smaller timescales than τ might represent an artifact due to thermal fluctuations rather than representing a true mode of motion [78]. Over the years, tICA has become a popular tool for extracting kinetically relevant CVs in the community of Markov state models (MSM) analysis [79,80,81,82]. Specifically, it has been proven that tICA can provide an optimal approximation of the true eigenvectors of the Markov transition matrix [83,84]. In practice, tICA is employed at the very beginning of MSM construction to map the usually high-dimensional input features into a lower-dimensional space that captures the relevant dynamics of the system, on which the subsequent clustering to identify the microstates is then performed. Moreover, it has been recently shown that tICA can be successfully used as CVs for MD/ML resampling (tICA-Metadynamics) even in a low-data regime [85]. A non-linear extension obtained through a Gaussian kernel (landmark kernel tICA-metadynamics) has also been proposed [85].

#### 2.1.4. Neural Networks and Deep Learning

**Figure 5:**
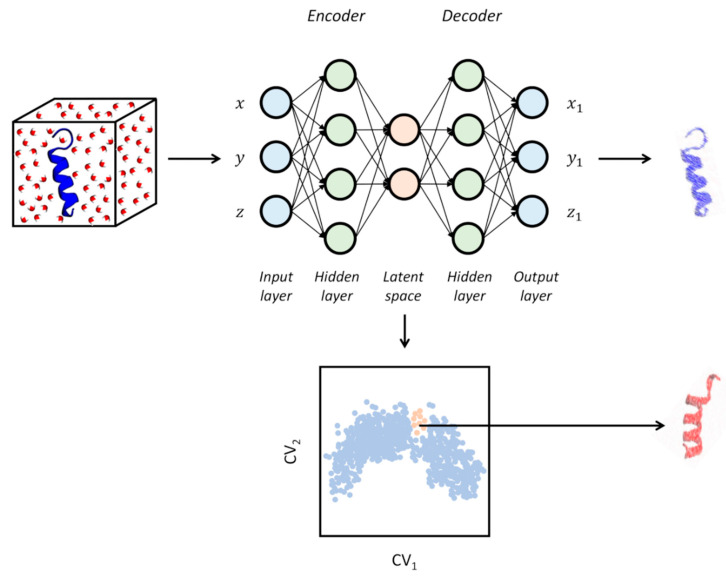
Schematic representation of an autoencoder. Basing on the conformations sampled through the MD simulations (protein in blue ribbons with surrounding water molecules), a latent space can be learned and trained (blue dots in the bottom plot) in a way to reproduce at best the original input data structure (blurred blue protein on the right). The latent space information can also be used to generate previously unexplored conformations (red dots in the bottom plot and red protein on the right).

During the last couple of years, active research in the field of dimensionality reduction for analyzing simulation data has mostly been focused on the investigation of the potential offered by deep learning methods like neural networks (NN). Among these, auto-associative neural networks (ANNs, or autoencoders) represent a class of unsupervised ML methods based on the sequential use of two NNs. The first network is used for encoding the low-d embedding (often called the “latent space” in this context) from the input features, while the second takes care of decoding the compressed information of the latent space for reconstructing the original high-dimensionality (see Figure 5) [86]. Each network is composed of a layer of “neurons”, whose activation is defined as

(10) $$h_{i}=f\left(w_{i}x+b_{i}\right)=f\left(\sum_{j=1}^{N}w_{ij}x_{j}+b_{i}\right),$$

where f is a non-linear activating function (a sigmoid function), x is the input vector, $w_{ij}$ are the elements of the weight matrix of the layer, and $b_{i}$ is the biases of the layer. A crucial advantage of autoencoders over the methods discussed in the previous paragraph is that the difference among the original high-d space of data and the reconstructed version of it can be used as a direct measure of the performance of the ML method. In this way, autoencoders can be trained to obtain an optimal non-linear low-d embedding. From a practical standpoint, training the network corresponds to optimizing weights and biases to minimize the reconstruction error through iterative procedures where each minimization step is referred to as an “epoch”. To control the magnitude of weights and biases during training, regularization terms are usually considered [86].

As previously noted, this is a field that is rapidly evolving. However, without claiming exhaustiveness, we can identify two major classes of applications of DL in the context of MD simulations, even though there are no conceptual boundaries between them, and overlaps can be envisioned. The first focuses on the unsupervised extraction of statistically relevant information like the equilibrium population of states with a particular emphasis on the estimation of rate constants. As we discussed in the previous chapter, a “kinetically relevant” low-d embedding is instrumental to this aim. The second group of methods is more oriented to the automatic extraction of relevant CVs for on-the-fly MD/ML sampling or later use. In both cases, a mechanistic interpretation of the events that occurred during the simulation is also guaranteed. Time-lagged autoencoders like TAE, which extends the domain applicability of autoencoders to the modeling of time-series data, fall in the first class of methods [87]. Similarly, the variational dynamics encoder (VDE) is able to capture the relevant dynamics of complex processes through a non-linear embedding by adding Gaussian noise regularization (the so-called variational autoencoder, VAE) [88]. Closely related to these methods, the main goal of VAMPnets is rather to replace the well-established pipeline of tICA extraction, clustering, and MSM kinetic model building through a fully automated deep neural network, relieving the user from subjective choices and error-prone steps [89].

Concerning the methods focused on CV discovery, we note that the low-d space obtained through autoencoders is by construction a differentiable function of the input coordinates and it is devoided from the out-of-sample problem, making this class of ML methods intrinsically superior over conventional dimensionality reduction methods for CV extraction, MD/ML resampling, and even for on-the-fly MD/ML sampling. Molecular enhanced sampling with autoencoders (MESA) developed by Chen and Ferguson is based on successive rounds of non-linear CV discovery and biased sampling of these CVs [90,91]. Specifically, MESA is an on-the-fly MD/ML-guided sampling protocol which can be summarized as follows: generation of initial training data through previous unbiased or biased MD simulations, autoencoder-based CV discovery, boundary detection for identifying unexplored regions of the CV space, enhanced sampling in the low-d embedding, convergence assessment, and, finally, free energy estimation. A similar procedure is exploited in the reweighted autoencoded variational Bayes for enhanced sampling (RAVE) method proposed by Tiwary and coworkers [92]. Conversely, EncoderMap is an NN method developed by Lemke and Peter which combines the advantages of autoencoders with the loss function employed by Sketch Map (Equation (6)) to get a better defined and interpretable low-d embedding [93]. Remarkably, EncoderMap takes full advantage of the potentialities offered by autoencoders, as it not only allows to obtain a differentiable function mapping from the high- to the low-d space, but it can also be used for backward mapping. From this standpoint, the mapping function linking the low-d embedding to the original high-d space can be used to generate previously uncharted molecular configurations. As the authors stated, this unique feature provided by this class of methods can be used as a new type of molecular modeling. This possibility was further investigated in the improved variant EncoderMap(II) implementing the ability to reproduce both short-ranged and long-ranged features, which is essential for preserving chemical accuracy in the generation of conformations for large and even multi-domain proteins [94]. From a different standpoint, it is worth mentioning the release of the python package named Anncolvar from Spiwok and coworkers [95]. This package allows the training of a neural network for CV extraction and resampling within the PLUMED program [23]. To the best of our knowledge, this is the first example of the implementation of an autoencoder specifically designed for its use in the field of biomolecular simulations and with an eye to the community of researchers using enhanced sampling. This further underscores the importance and popularity gained by these ML methods in the context of MD simulations.

We wish to conclude this section on DL methods with a word of caution. Like clearly stated by Sultan et al., care must be taken when using autoencoders for analyzing MD trajectories, as their superior performance compared to more conventional dimensionality reduction methods is not free from potential pitfalls [96]. Most importantly, the black-box nature of NNs makes it hard to understand what the autoencoder actually learns, potentially leading to identifying a low-d embedding that cannot necessarily be considered a good CV for biased MD/ML resampling [96]. It is also true, however, that some of the above-reported methods are specifically designed to include the information of the dynamics, and therefore this risk should be contained. In their work, Sultan et al. introduced a tICA-VDE extension that is optimally suited to extract relevant and transferable CVs [96].

### 2.2. Supervised Learning Methods

**Figure 6:**
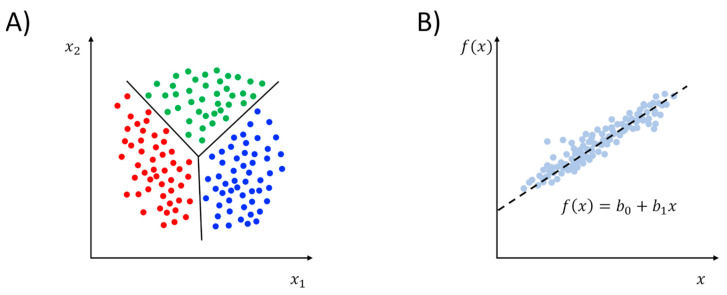
Pictorial representation of supervised learning class of methods: classification (panel (**A**), linear discriminant analysis (LDA) is displayed as an example) and regression (panel (**B**), linear regression displayed as an example,).

Compared to the plethora of unsupervised learning methods described in Section 2.1, the use of supervised ML methods for analyzing and biasing MD simulations is much more limited in the literature. Typically, the tasks that are considered by supervised learning can be distinguished in regression and classification (see Figure 6). Regression problems deal with the construction of quantitative predictive models relating some continuous dependent variables to the independent variables. In this case, ML methods are used to quantify such a relationship provided that a linear or non-linear model function is supplied by the user. Conversely, classification problems deal with the construction of qualitative predictive models able to predict the categorical class labels for a given observation. Some supervised ML methods, such as decision trees and ANN, can be used for both classification and regression problems with opportune measures. Other algorithms, such as linear regression for regression problems or logistic regression for classification problems, cannot easily be exploited for both types of tasks.

The use of regression in the context of the analysis of MD trajectories has been pioneered by Hub and de Groot [97]. The authors observed that, in the context of biomolecular simulations, dimensionality reduction is often carried out for getting mechanistic insight into the system under investigation. This is especially relevant in the case of proteins that are typically known to achieve their biological function, like catalysis, gating, and signal transduction, among others, through collective atomic motions. When dimensionality reduction is performed with well-established methods like PCA, however, the collective motions that one gets are by construction the widest ones, but they are not necessarily directly involved in the biological function [97]. This relation between function and motion is achieved by introducing a functional quantity f so that for each frame of the trajectory it can return a single value. This functional quantity can be any observable that might be relevant to describe the function one wishes to characterize, like atomic distances, binding sites’ volume, solvent-accessible surfaces, and so on [97]. Then, assuming that f is a linear function of PCs, through a least-squares optimization procedure, a quantitative model of the observable as a function of the PCs can be obtained much like in PC regression (PCR). This corresponds to maximizing the Pearson’s correlation coefficient, but the MI can also be maximized to include the non-linear dependency of f as a function of atomic coordinates. The vectors that the method finds out are a linear combination of PCs, and the one displaying the largest correlation with the given observable is referred to as maximally correlated motion (MCM). However, as the authors pointed out, this vector is unaware of the underlying free energy landscape, therefore a sort of correction is also devised in order to obtain a physically meaningful coordinate that represents the most probable collective motion that determines the MCM (ensemble-weighted MCM, ewMCM) [97]. In order to avoid overfitting, a cross-validation procedure is implemented which envisions the partition of the trajectory into a training set and a test set. The method is called functional mode analysis (FMA), and the MCM/ewMCM can be also used for MD/ML resampling through biased simulations [97]. The initial assumption that deviations in f are mostly determined by PCs was later weakened in a generalization of the method (partial least-squares FMA, PLS-FMA) that simultaneously optimizes both the model and the basis vectors, yielding to more robust models with a substantially smaller number of components [98]. PLS-FMA was tested on the T4 lysozyme and Trp cage, and then applied to the yeast and human aquaporin channels (Aqy1 and AQP1, respectively), and the CLC-ec1 chloride antiporter using the active site geometry, hydrophobic solvent-accessible surface, channel gating, water permeability, and dihedral angles as functional observables [98]. Very recently, PLS-FMA has been used to identify allosteric communication pathways between the activation gate and the selectivity filter of potassium channels [99].

Whenever one wishes to identify motions involved in the discrimination between states, rather than determining the variation on a continuous observable, classification methods are more suitable than regression. One such method, linear discriminant analysis (LDA), seeks to identify the optimal hyperplane separating the two or more groups of data (that, unlike in cluster analysis, must be known a priori). Accordingly, partial least-squares LDA (PLSA-DA) was used by Peters and de Groot to analyze a series of bound and unbound ubiquitin complexes [100]. By labeling the trajectories according to the binding state (−1 for unbound, +1 for bound), they trained a model returning a vector which maximized the difference of the projection of structures from different classes while minimizing the difference from the same class. By doing so, they observed that the conformations accessible to the bound ubiquitin were partially overlapping those of the unbound ubiquitin, suggesting that conformational selection was the preferred recognition mechanism over induced-fit [100]. The linear discriminant analysis with ITERative procedure (LDA-ITER) is another method specifically designed to overcome a potential bias affecting PLS-DA and related to the fact that the projection vector is obtained through the averaged structures belonging to the two classes, making the results strongly dependent on the anisotropy of the investigated proteins [101].

LDA has also been recently used to approach the problem of identifying optimal CVs for biased MD/ML resampling. In particular, this class of supervised ML can be used to train CVs in the special case of previous knowledge of the end states. From this standpoint, the following methods resemble in spirit the already mentioned path CV framework [66]. A variant of LDA called harmonic LDA (HLDA) has been indeed recently introduced by Parrinello and coworkers as a CV suited to distinguish between two metastable states. This can be derived from short unbiased MD simulations initiated in the end states and through a series of features for mapping these states in a high-d space [102]. The method was further generalized to a multiclass problem (MC-HLDA) in order to treat more than two states simultaneously [103]. The obtained CV space was proven to be effective for reconstructing the free energy surface of chemical reactions through metadynamics, but was unable to lead to a converged free energy surface when a more complex problem like the folding of chignolin was considered [104]. Finally, we mention that other supervised ML methods other than LDA (like support vector machines, and logistic regression) have also been employed for the automatic detection of CVs for MD/ML resampling [105].

## 3. Learning from Molecular Dynamics Trajectories and Experimental Data

As hardware capacity advances and methods to improve sampling are optimized, the observation of biomolecular events on biologically relevant timescales is gradually becoming more accessible [10,106]. Related to this, possible limitations of the empirical models underlying MD, i.e., the empirical force fields, also become more evident as a result. In fact, deficiencies can appear when a meaningful comparison with reference experimental data is conducted. However, such comparison may display disagreement. While possible limitations in the model become apparent, we can however make optimal use of the available experimental knowledge to improve the quality of the simulations and account for deficiencies thereof. Indeed, the combination of experimental and theoretical sources of information is emerging as an effective strategy to get insights into the structural and functional features of biomolecules [107,108,109]. As a final note, we highlight that failures are typically ascribed to the MD simulations since they are the result of an empirical model. While this is undoubtedly reasonable, it is nevertheless advisable to include experimental information with criticism, as, in general, any source of data can be affected by errors of systematic, statistical, and procedural nature.

### 3.1. Validating MD Simulations through Comparison with Experiments

**Figure 7:**
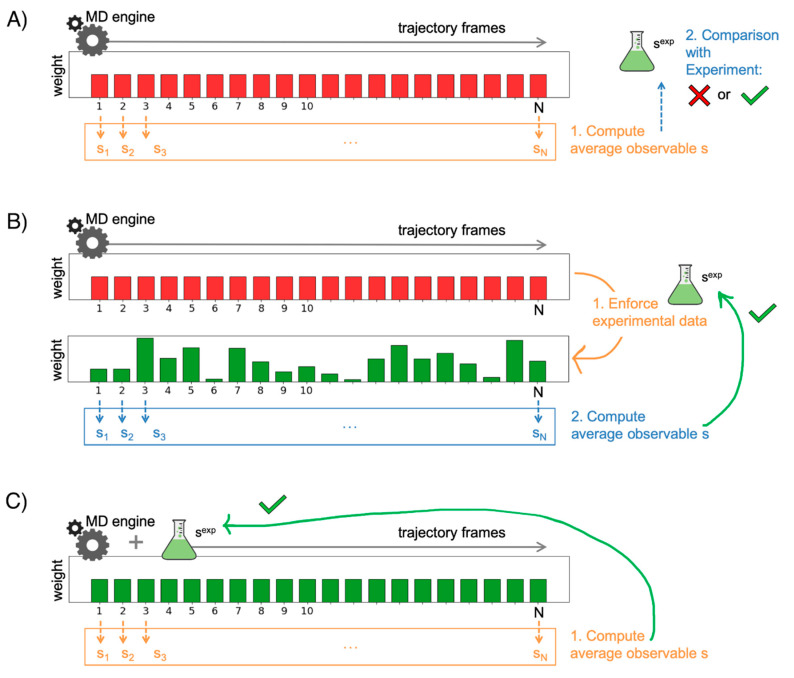
Using MD simulations in combination with experimental information. (**A**) Through a validation procedure, it is possible to estimate the agreement between computed quantities (average observable s, in the figure) and reference experimental data (s^exp^). (**B**) Correction of the sampled data through a reweighting procedure can improve the agreement between predicted (MD trajectory) and measured (experiments). (**C**) Enforcing the experimental information in an on-the-fly fashion, the sampled ensemble is restrained to best match the experimental one.

A typical pipeline when exploiting MD is to first set up and carry out the simulations of the biomolecular system, then analyze the resulting trajectories to compute relevant quantities of interest, and finally perform a comparison of the computed outcomes with a measure obtained experimentally. In such a way, what we do is to validate the results of the simulations against reference experimental data. To ensure a fair and meaningful comparison, it is essential that the MD runs are performed under conditions that are as close as possible to those in which the experimental measures were conducted. In this respect, for instance, it was widely reported over the years how ionic conditions of the bulk, both in terms of ionic nature and strength, can play a critical role when biomolecular processes are investigated [110,111,112,113]. With such considerations taken into account, the validation procedure reports on to what extent the empirical model possibly succeeds, or fails, in generating realistic and reliable results. Figure 7A depicts the fairly simple principle behind this routine procedure. For each snapshot of the MD trajectory generated by the simulation of a biomolecular system, we compute an observable of interest that we wish to compare with the experiments. A natural choice would be, among all the predicted values from the MD simulation, to pick the ones that provide the best match. The corresponding MD snapshots would thus represent the system configurations that best reproduce the reference experimental data. However, the experimental measure is most times conducted on heterogeneous systems, as the equilibrium population typically comprises a variety of different states of the biomolecule. As a result, the measurement does not reflect a single configuration but is instead an average over the whole ensemble of states. This is true for popular methods that are routinely employed to validate simulation data such as nuclear magnetic resonance (NMR) [114,115,116], small-angle X-ray scattering (SAXS) [117], double electron–electron resonance (DEER) [118], and Förster resonance energy transfer (FRET) [119]. Therefore, in order to pursue a meaningful validation, one should first average the observable value over all the MD trajectory frames and then compare this result with the reference experimental one.

### 3.2. Improving the Agreement through Ensemble Reweighting

If the validation procedure reports a disagreement between predicted (from the MD runs) and measured (in the experiments), one can still make optimal use of the available data through a reweighting strategy. In particular, the MD trajectory frames can be re-assigned suitable weights that allow improving the agreement with the experiments. The procedure is illustrated in Figure 7B. In this scheme, the configurations which are assigned higher weights are those that will contribute more to the final average, that is to the predicted value for the observable. In other words, such structures will be more representative of the experimental ensemble. As a result, the inaccuracies deriving from the employed empirical force field are corrected by including the guidance of the experimental data, thus improving the agreement. The use of the maximum entropy principle [120,121] is gaining popularity to implement such a strategy [19,122]. Specifically, given an initial (prior) distribution, by exploiting the maximum entropy principle, it is possible to identify a new distribution (posterior) that is as close as possible to the original one and matches the experimental reference data. In other words, the initial distribution is subject to the least possible perturbation that allows improving the agreement with experimental observation. Under the maximum entropy framework, the new distribution can be represented as

(11) $$P_{ME}\left(x\right)\propto e^{-\lambda \left(s\left(x\right)\right)}P_{0}\left(x\right).$$

This form gives all the possible posterior distributions *P_ME_* which are the closest to the prior distribution *P*_0_. Here, *s*(**x**) is the observable of interest computed for each configuration **x** sampled in the MD simulations. Among the posterior distributions *P_ME_*, the one that gives the best match with the experiment is sought. Such a latter requirement can be fulfilled by solving a minimization problem aimed at identifying the suitable value of the parameter λ [19]. This reweighting framework based on the maximum entropy principle has shown to be effective when applied to biomolecular systems of a different kind [123,124,125,126,127,128]. The procedure has been exploited by Bottaro et al. to reconstruct the conformational ensemble of four model tetranucleotides using extensive atomistic MD simulations and NMR experimental data [123]. Employing the simulation data alone, a significant disagreement with respect to the experiments was apparent. Thus, the simulated ensemble was refined through reweighting using the NMR experimental data, including nuclear Overhauser effect (NOE) intensities and scalar couplings, in order to improve the agreement. A similar pipeline using NOEs experimental data to refine atomistic MD simulations was also pursued on a longer RNA construct, of 29-nucleotide length, belonging to the SINEUP family [126]. Other applications using NMR data were conducted on a nonapeptide, where simulated ensembles via MD were used in conjunction with ^3^J coupling and RDC data [127], and on an intrinsically disordered protein, where coarse-grained simulations were reweighted with RDC measurements [125]. Reweighting using hydrogen–deuterium exchange combined with mass spectrometry (HDX-MS) data was also considered by Bradshaw et al. [128]. The scheme was first explored using artificial HDX-MS representing a conformational ensemble of the periplasmic binding protein TeaA that rapidly interconverted between its open and closed states. Such data were used to reweight bias-exchange metadynamics simulations. The procedure was then applied to the amino acid transporter LeuT membrane protein using experimental HDX-MS data. Różycki et al. included information from SAXS measurements performed at high-salt and low-salt concentrations to reweight coarse-grained simulations of CHMP3, a key protein of the ESCRT protein assembly [124]. As a result of the procedure, further insights into the conformations adopted by CHMP3 in its activated and autoinhibited states were obtained [124].

Noteworthy, the reweighting procedure is carried out as an analysis on outcome trajectories of MD simulations, i.e., after the MD runs are performed. Thus, a striking advantage of this procedure is that it can be repeated using additional experimental data, or different ones, with no need of performing new simulations. A further aspect is the possibility to include a regularization term, that can be introduced in the expression to model the experimental error and other sources of possible errors [19]. Indeed, in this respect, we note that the forward model, which is the form through which the observable is computed from the MD snapshots, can also incorporate errors. The effect of including such regularization, which the ML community is familiar with, is to soften the restraint towards the experimental reference. Thus, while the average computed after applying the reweighting procedure is going to match the experimental reference by construction, the application of a reweighting procedure with a regularization term is going to result in a computed average that sits between the one predicted from the prior with no reweighting and the experimental one. Finally, we note that the approach is not devoid of limitations. In particular, for a meaningful reweighting to be applicable, a certain degree of overlap is required between the sampled conformations and those comprised in the experimental ensemble, which is not the case when the domain sampled by the MD simulations contains a scarce number of configurations consistent with the experiment, if at all [129,130]. In such cases, the inapplicability of the reweighting procedure becomes apparent as either a large fraction of the total weight is assigned to one or few frames, or the minimization is not able to converge to a suitable *λ*. An illustrative example of this issue in a one-dimensional model is provided in the insightful review by Cesari et al. [19].

### 3.3. Enforcing Experimental Information during the Simulations

Another option that exploits the maximum entropy strategy and that in principle avoids the just-mentioned condition consists of including the experimental knowledge during the MD simulations [19,131]. In practice, the ensemble average computed from the simulations is enforced to match the experimental one in an on-the-fly fashion. This is achieved by modifying the system potential energy through the inclusion of an additional term, which has the effect of constraining the system towards configurations in better agreement with the reference experimental data [132,133]. A schematic depiction is given in Figure 7C. While, in principle, such a procedure has the advantage of producing a sampling domain which is, by construction, more consistent with the reference data, this nevertheless implies having chosen the target average value for the observable a priori, before starting the simulation. In other words, in the case where additional or different experimental data become available, within this framework, a new simulation needs to be performed from scratch. The approach was successfully applied to RNA nucleosides and dinucleotides to enforce NMR experimental data during replica-exchange MD simulations [132]. In particular, the information from ^3^J scalar couplings was exploited to guide the enhanced sampling simulations. Force field corrections to match the experimental reference were thus identified and were then validated over independent NMR solution experiments.

A different approach with the same purpose of instructing MD simulations by taking advantage of experimental knowledge is based on a multi-replica strategy [134,135,136]. Specifically, multiple replicas of the system are simulated with MD at the same time. Then, the average of the interesting observable over such replicas is enforced to match the experimental value. As a result, a restrained ensemble is obtained. Notably, in the limit of a large number of replicas employed, the method has been shown to produce the same ensemble of configurations like the one generated through the maximum entropy scheme used on-the-fly [131,137,138]. The multi-replica strategy was used by Best and Vendruscolo to generate an ensemble of structures of the third fibronectin type III domain from human tenascin that was consistent with available NMR data [135]. Similarly, the conformational variability of the ubiquitin protein in solution was probed using MD simulations and enforcing experimental information from NMR relaxation experiments using the multi-replica approach [136]. A more recent study relying on the use of multiple replicas was conducted by Hermann and Hub, where the strategy was used to enforce SAXS experimental data in an on-the-fly fashion during MD simulations of intrinsically disordered proteins [139].

Finally, inspired by this replica approach, the metainference method was further devised [140]. Metainference combines the mentioned multi-replica scheme with Bayesian inference. The inclusion of the statistical basis of the latter allows one to tune the strength of the restraints towards the reference experimental data. In such a way, all possible sources of errors, including errors in the experimental data or in the forward model, are taken into account. The method and its declination where metainference is combined with metadynamics (metadynamic metainference) [141] have been applied to diverse biological systems and take advantage of different sources of experimental information. Heller et al. studied the binding of the small molecule ligand 10058-F4 to the disordered protein c-Myc using metadynamic metainference simulations with experimental restraining consisting of NMR data, specifically backbone chemical shifts [142]. In a similar biological context, metadynamic metainference and NMR chemical shifts were exploited by Hultqvist et al. to get insights into the interaction of the two disordered proteins CID and NCBD [143]. Backbone chemical shifts were also used by Buckle et al. to investigate the interaction of the SNa15 peptide with non-native mineral surfaces [144]. Finally, concerning NMR, RDC data were employed in the metainference framework by Weber and coworkers to study the conformational space accessible to the LC protein [145]. Interestingly, cryo-EM experimental data were demonstrated to suitable to be integrated in a metainference scheme. In particular, Bonomi and coworkers took advantage of cryo-EM information in the effort of characterizing the structure and dynamics of the integral membrane receptor STRA6 [146]. Another example was reported by Vahidi et al. in their investigation on the structural dynamics of the ClpP proteolytic complex, where cryo-EM data were used to perform metainference [147]. Finally, SAXS intensities were also explored as a source of experimental information to be used with metainference. Paissoni and coworkers exploited this strategy to investigate the conformational ensemble of K63-linked diubiquitin [148] and to refine models of nucleic acid-protein complexes [149]. Similarly, Kooshapur et al. used SAXS experimental data in a metainference framework to derive a structural model of a complex between an RNA-binding protein and a microRNA [150].

## 4. Conclusions and Perspectives

In this review, we have summarized the currently available strategies that can be exploited to make optimal use of a constantly increasing volume of data in the field of molecular dynamics simulations applied to pharmaceutically relevant biological systems. Specifically, we have shown that this wealth of data can either be the output of MD simulations or come from experimental sources. The available information can be then exploited to inform subsequent calculations or to improve the prediction of relevant observables. We have also shown that some of the most popular analysis tools that have historically been employed in the field of MD simulations pertain to the domain of machine learning methods, and we have provided an overview of the most influential approaches belonging to the classes of supervised and unsupervised ML methods. For each considered approach, relevant examples of applications in the field of biomolecular simulations, and more specifically to drug design, have been briefly discussed when available. Finally, we have summarized the simulative and analysis approaches that exploit experimental knowledge to improve the quality of computational predictions.

In summary, we have shown how the increasing richness of data (up to the regime of big data) is prompting a shift in the methodologies employed in the field of molecular dynamics in favor of more automatized and less human-dependent procedures. This has started to change not only the way we are analyzing but also the way we are conceiving MD simulations as a whole. In fact, the boundary between data production (i.e., the trajectory above all) and data analysis, that have traditionally been considered as separated processes (see Figure 1, path “1”), is getting less sharp in the newest MD/ML implementations (path “2” in the same figure). Similarly, but from a different perspective, experimental data, which are usually only considered during force field development, are gaining a key role in post-processing or even in guiding MD simulations (Figure 1, path “3”). These advances will ultimately lead to more efficient and/or predictive data-driven MD simulations with important implications for the entire community of biological simulations, including applications in the field of computational drug discovery.

## Figures and Tables

**Table 1:** Classification of the most popular ML algorithms.

Class	Learning Task	Method
**Supervised Learning**	Regression	Linear regression ^1^ Non-linear regression Support vector regression (SVR) Artificial neural network (ANN)
	Classification	Logistic regression (LR) ^1^ Linear discriminant analysis (LDA) ^1^ Support vector machines (SVR) k-nearest neighbor (kNN) Decision trees/random forests Artificial neural network (ANN)
**Unsupervised Learning**	Clustering	Hierarchical agglomerative/divisive k-means/-medoid Gaussian mixture models (GMM) ^1^ Density-based (DBSCAN) Self-organizing maps (SOM)
	Dimensionality Reduction	Principal component analysis (PCA) ^1^ Kernel-PCA (kPCA) ^1^ Independent component analysis (ICA) ^1^ Multidimensional scaling (MDS) ^1^ Isometric feature mapping (IsoMap) ^1^ Locally linear embedding (LLE) Diffusion maps (dMaps) ^1^ Artificial neural network (ANN) ^1^

^1^ Examples of this ML method have been described in the context of MD analysis and are reported in the text.
